# Effect of a phosphodiesterase-5A (*PDE5A*) gene polymorphism on response to sildenafil therapy in canine pulmonary hypertension

**DOI:** 10.1038/s41598-019-43318-z

**Published:** 2019-05-03

**Authors:** Yu Ueda, Lynelle R. Johnson, Eric S. Ontiveros, Lance C. Visser, Catherine T. Gunther-Harrington, Joshua A. Stern

**Affiliations:** Department of Medicine and Epidemiology, School of Veterinary Medicine, University of California-Davis, One Shields Avenue, Davis, CA 95616 USA

**Keywords:** Genetics research, Hypertension, Respiratory tract diseases, Cardiovascular genetics

## Abstract

Pulmonary hypertension (PH) is a common clinical condition associated with morbidity and mortality in both humans and dogs. Sildenafil, a phosphodiesterase-5 (PDE5) inhibitor causing accumulation of cGMP, is frequently used for treatment of PH. The authors previously reported a *PDE5A*:E90K polymorphism in dogs that results in lower basal cyclic guanosine monophosphate (cGMP) concentrations than in wild-type dogs, which could contribute to variability in the efficacy of sildenafil. In this study, response to sildenafil therapy was evaluated in dogs with PH by comparing echocardiographic parameters, quality-of-life (QOL) score, and plasma cGMP concentrations before and after sildenafil therapy. Overall, tricuspid regurgitation estimated systolic pressure gradient (PG) and QOL score were significantly improved after sildenafil therapy, and the plasma cGMP concentration was significantly decreased. Dogs that had a heterozygous *PDE5A* status had a significantly worse QOL score when compared to the wildtype group after sildenafil treatment. The simple and multiple regression analyses revealed a significant but weak prediction for the percent reduction in QOL score with sildenafil treatment by plasma cGMP level and by the *PDE5A*:E90K polymorphic status. This study showed that sildenafil treatment improved PH in dogs, and the *PDE5A*:E90K polymorphism blunted the efficacy of sildenafil in terms of QOL improvement.

## Introduction

Pulmonary hypertension (PH) is a pathologically complex condition characterized by abnormally increased pulmonary arterial (PA) pressure. PH is generally categorized by the underlying cause including pulmonary parenchymal or interstitial lung disease, pulmonary thromboembolism, cardiac disease, or idiopathic etiologies^1–5^. These underlying disorders result in elevated PA pressure by multiple mechanisms including elevations of pulmonary venous pressure, increased pulmonary vascular resistance, and altered pulmonary blood flow^2^. Without prompt treatment of PH and its underlying condition, PH often induces further vasoconstriction, vascular smooth muscle proliferation, and microthrombus formation in the pulmonary vasculature leading to worsening of PH and ultimately right-sided heart failure^6,7^.

Sildenafil is an orally active selective PDE5 inhibitor that has been used for treating PH in humans and other species including dogs^8–13^. The main beneficial effect of sildenafil in patients with PH is believed to result from accumulation of cGMP in the pulmonary vascular smooth muscle due to inhibition of cGMP catabolism^14–17^. Sildenafil has further been demonstrated to decrease PA resistance by preventing PA wall proliferation^18,19^. Sildenafil effects are not limited to the pulmonary circulation as it also increases the cGMP concentration in the myocardium and thus may enhance cardiac function and prevent hypertrophy in mice with chronic pressure overload^20^. In dogs, there are several studies investigated the response to sildenafil therapy in experimentally-induced and naturally-occurring PH^12,13,21–25^. These experimental models of PH in dogs were induced either by hypoxic pulmonary vasoconstriction or pulmonary thromboembolism^23,25–27^. These experiments reported that short-term sildenafil therapy significantly decreased mean PA pressure. Several small prospective and retrospective studies with naturally-occurring PH in dogs have also documented significantly reduced PA pressure in response to short and long-term sildenafil therapy, but one study reported no significant PA pressure reduction with long-term sildenafil therapy leading to some contradiction of findings in the clinical setting^12,13,22,24^. In human patients with pulmonary hypertension, long-term sildenafil therapy has been considered safe and effective^9,10,28,29^. However, several complications with chronic high-dose usage of sildenafil have been reported. These side effects include tachycardia, dyspepsia, headache, and visual disturbance^30–34^. These are generally believed to be due to vasodilatory effects resulting from PDE5 inhibition. In dogs, long-term sildenafil is considered safe, but a large prospective study has not yet documented the frequency of adverse events in dogs with long-term sildenafil therapy.

In addition, significant inter-individual variability in the response to sildenafil has been reported in humans and dogs^35–37^. The intrinsic and extrinsic causes of inter-individual variability include poor owner compliance, drug-to-drug interactions, and upregulation or downregulation of other mechanisms, and possible pharmacogenetic impacts. Several genetic polymorphisms have been identified as associated with altered efficacy of sildenafil in humans^38–41^ including variants in *PDE5A*^42^. The *PDE5A* gene and its promoter regions have been sequenced and the functional regions are well described in humans^43–45^. The presence of polymorphisms in *PDE5A* in humans has been linked to reduced response to nitric oxide and altered levels of cGMP^8,46^. Alternation of the coding sequence in *PDE5A* was also demonstrated to affect the binding affinity of phosphodiesterase inhibitors^47^. In dogs, a *PDE5A* gene polymorphism substituting a glutamic acid for lysine at the 90^th^ amino acid (*PDE5A*:E90K) has been identified, and the homozygous polymorphism status was reported to associate with lower basal cGMP concentrations compared to wildtype dogs^48^. However, no studies have been performed to investigate the impact of the *PDE5A*:E90K polymorphism on the response to sildenafil therapy in dogs with PH. Understanding the functional role of identified *PDE5A* gene polymorphisms is key to understanding their clinical relevance.

In this study, we first aimed to confirm the quantitative and qualitative effect of long-term sildenafil therapy on dogs with naturally-occurring moderate or severe PH. We hypothesized that long-term sildenafil therapy significantly reduces severity of PH, improves QOL score, and increases circulating cGMP concentrations in dogs with naturally-occurring moderate to severe PH. Our second aim was to determine the effect of the *PDE5A*:E90K polymorphism on sildenafil efficacy in dogs with PH. We hypothesized that the presence of the *PDE5A*:E90K polymorphism reduces response to therapy with sildenafil in cases of moderate to severe naturally-occurring PH. To test these hypotheses, selected echocardiographic parameters, quality of life questionnaire, and plasma cGMP concentrations were obtained from dogs with moderate to severe PH before and after sildenafil therapy and these measures were compared in dogs genotyped to have varied *PDE5A* genetic status. These findings will help establish the value of a possible canine model of naturally-occurring PH and determine the impact of the *PDE5A* gene mutation on the efficacy of sildenafil therapy.

## Results

### Animal characteristics

Forty-one dogs met the inclusion criteria and were enrolled in this study to completion. Of these, 8 dogs were Chihuahua, 5 were mixed breed, 4 each were Shih Tzu and West Highland White Terrier, 3 each were Jack Russell Terrier and Pomeranian, 2 each were Maltese, Pekingese, Shetland Sheepdog, and one each were Affenpinscher, Cavalier King Charles Spaniel, Miniature Dachshund, Miniature Schnauzer, Papillon, Pug, Tibetan Terrier, and Toy Poodle. Seven (17.2%) dogs had PH of post-capillary etiology with or without other concurrent etiology of PH, whereas 34 (82.9%) dogs had PH of pre-capillary etiology. A total of 22 males (53.7%) and 19 females (46.3%) were enrolled. The median weight was 5.8 (IQR: 3.5–8.85) kg among all dogs with the median dose of sildenafil prescribed as 4.55 (IQR: 3.71–5.65) mg/kg/day overall. The mean age of all dogs was 11.5 (SD: ±3.65) years old. Systolic blood pressure was measured prior to enrollment to rule out systemic hypotension and hypertension, and median blood pressure was 125 mmHg (IQR: 115–146 mmHg) overall (Table 1).

**Table 1:** Baseline characteristics and mortality of dogs with different *PDE5A* polymorphic. status. The means (±SD) or medians (IQR) are shown in the table based on their normality distributions.

	Overall	Wildtype	Heterozygous	Homozygous	*P*-value
Sample number, n (%)	41	9	13	19	
Age (year)	11.5 (±3.65)	11.0 (±4.9)	11.2 (±4.6)	12.0 (±1.98)	0.72
Male, n (%)	22 (53.7)	2 (22.2)	9 (69.2)	11 (57.9)	0.083
Post-capillary etiology, n (%)	7 (17.2)	0 (0)	3 (23.1)	4 (44.4)	0.3
Weight (kg)	5.8 (3.5–8.85)	3 (2.34–6)	6.5 (3.8–9.05)	5.8 (4.3–9.6)	**0**.**033**
Sildenafil dose (mg/kg/day)	4.55 (3.71–5.65)	5.56 (3.13–6.71)	4.41 (3.4–5.82)	4.44 (4.05–5.26)	0.56
Systolic BP (mmHg)	125 (115–146)	112 (95–148.5)	143 (124.5–157.5)	124 (114–137)	0.064
Mortality, n (%)	12	2 (22.2)	3 (23.1)	7 (36.8)	0.61

BP; blood pressure.

**Figure 1:**
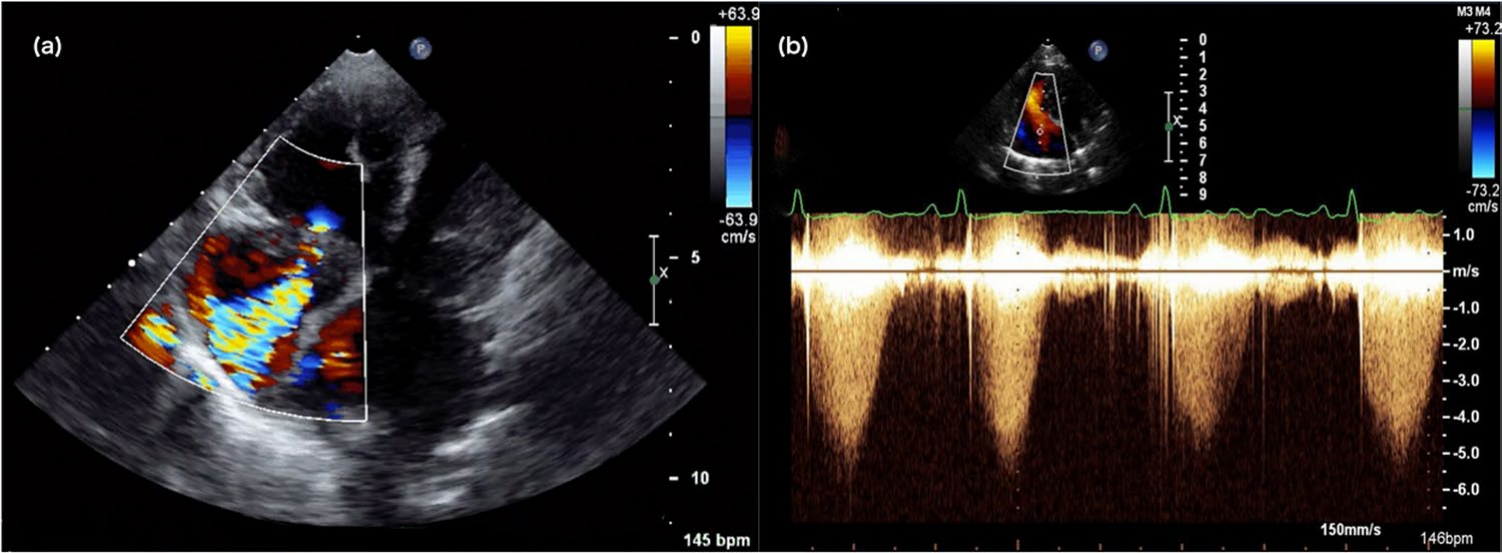
(**a**) A left apical four-chamber echocardiographic view of a dog with pulmonary hypertension shows color-flow Doppler of a severe tricuspid regurgitation jet. (**b**) A spectral Doppler image was used to measure the peak velocity of the tricuspid regurgitation. PG was calculated using the modified Bernoulli equation (PG = 4 × velocity^2^) without any addition for estimated right atrial pressure.

On echocardiographic examination prior to the sildenafil treatment, moderate to severe PH was diagnosed in all dogs enrolled (Fig. 1). Of these, 16 (39%) dogs were diagnosed with moderate PH and 25 (61%) dogs were diagnosed with severe PH. One dog showed signs consistent with right-sided congestive heart failure. A total of 28/41 (68.3%) dogs had septal flattening during diastole, systole or both diastole and systole.

**Figure 2:**
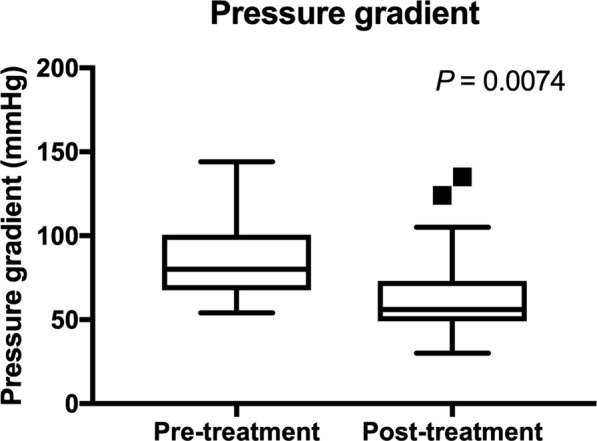
PG before and after sildenafil treatment is shown. The bars and boxes show the median and interquartile ranges. The whiskers represent 1.5 times the interquartile range and the black squares indicate outliers. There was a significant reduction of PG after sildenafil treatment (P < 0.0074).

**Figure 3:**
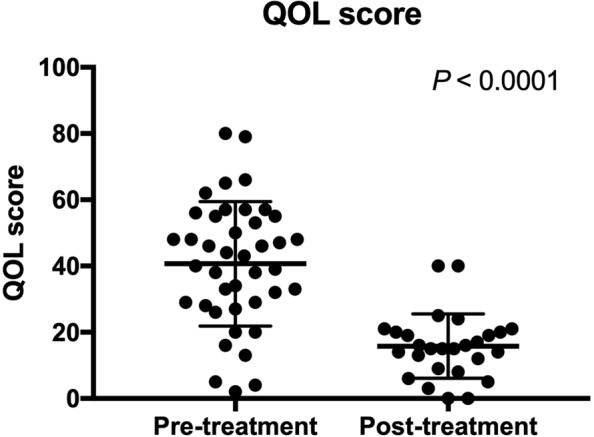
QOL scores before and after sildenafil treatment are shown. The bars show the mean and standard deviations. There was a significant reduction in the QOL score after sildenafil treatment (P < 0.0001).

**Figure 4:**
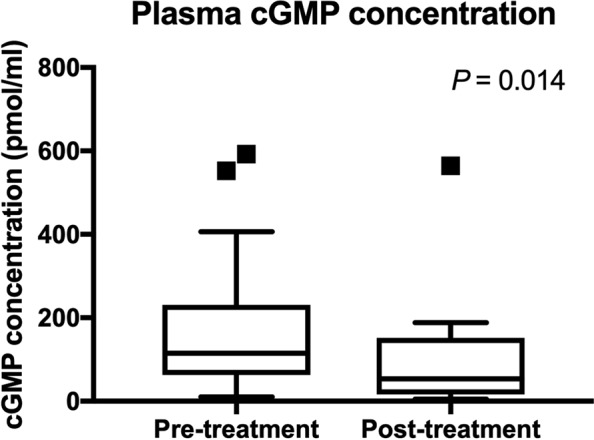
Plasma cGMP concentrations before and after sildenafil treatment are shown. The bars and boxes show the median and interquartile ranges. The whiskers represent 1.5 times the interquartile range, and the black squares indicate outliers. There was a significant reduction of plasma cGMP concentration after sildenafil treatment (P = 0.014).

A total of 12 dogs (29%) died or were euthanized prior to the opportunity for a reevaluation examination 25 to 35 days after initiating sildenafil therapy (Table 1). No obvious side effects of sildenafil therapy were reported by the owners, and death or euthanasia of these animals is considered to be due to progression of underlying disease conditions. Considering remaining dogs, there was a significant reduction of PG after sildenafil treatment (*P* = 0.0074) (Fig. 2). Other echocardiographic parameters including PVAT (*P* = 0.43), PVET (*P* = 0.44), and PVAT:ET (*P* = 0.94) were not significantly changed between pre- and post-treatment with sildenafil. The mean QOL score calculated based on the questionnaire was significantly reduced after sildenafil treatment, with a noted improvement (lower score) in QOL (P < 0.0001) (Fig. 3). Plasma cGMP concentrations were significantly reduced after sildenafil treatment (*P* = 0.014) (Fig. 4).

### *PDE5A*:E90K genotype

With regards to the *PDE5A*:E90K genotype, 9/41 (22.0%) dogs were wildtype, 13/41 (31.7%) were heterozygous, and 19/41 (46.3%) were homozygous. There were no significant differences in sex distribution, mean age, median body weight, etiology (pre- and post-capillary PH), and pre-treatment systemic blood pressure between the genotype groups (Table 1). There were significant differences in median body weight among genotype groups (*P* = 0.033) with lower body weight in the wildtype compared to the homozygous group (P = 0.041). There were no significant differences in the sildenafil dose among the genotype groups (Table 1). Of 12 dogs that died or were euthanized, two dogs were wildtype (2/9; 22.2%), three dogs were heterozygous (3/13; 23.1%), and seven dogs were homozygous (7/19; 36.8%). The mortality rates were not significantly different between these genotype groups (*P* = 0.61). When variant dogs were pooled, a Fisher’s exact test also revealed no statistically significant difference between the two groups and outcome (P = 0.7).

### Echocardiographic findings

**Figure 5:**
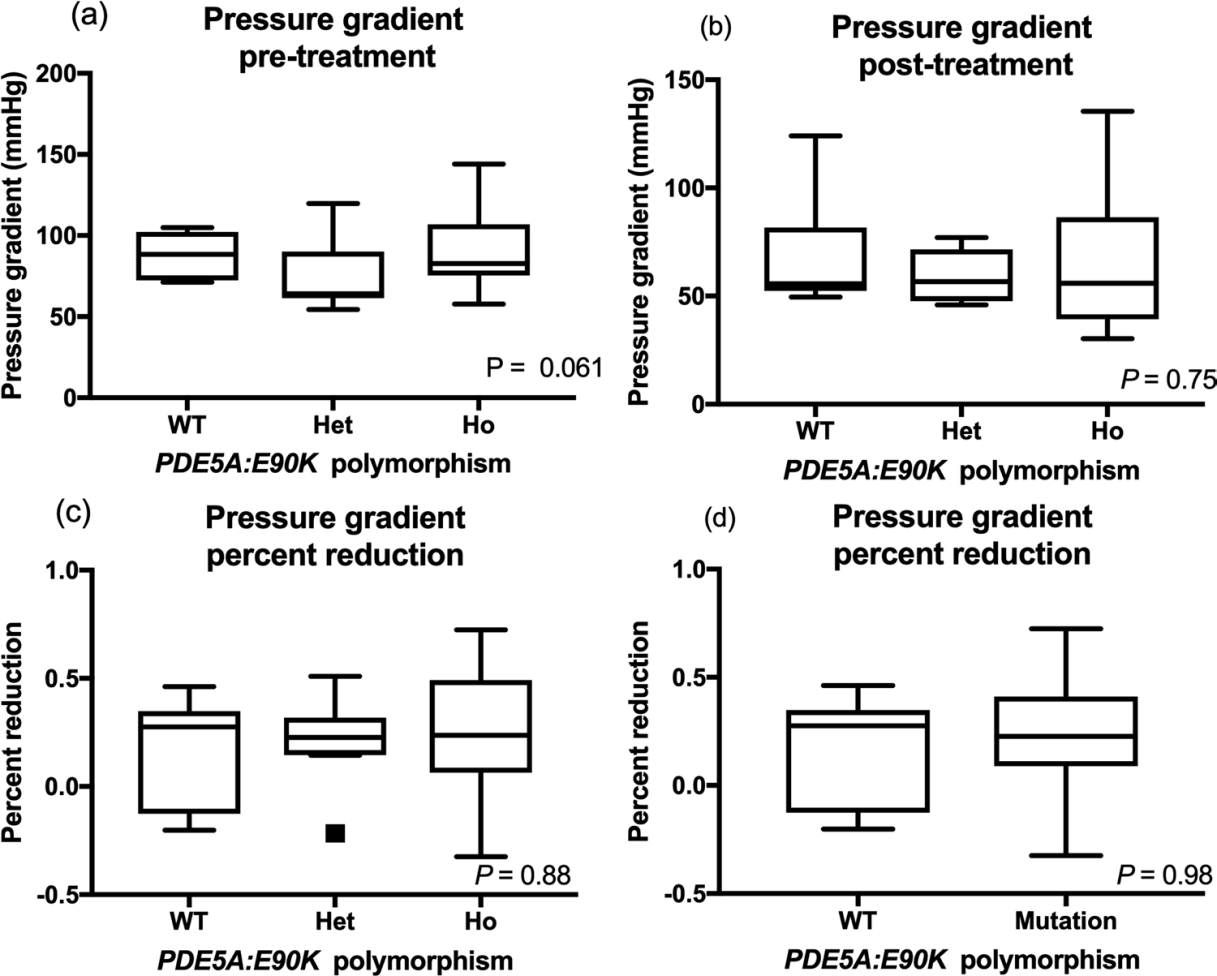
PG (**a**) before and (**b**) after sildenafil treatment was shown in the different *PDE5A*:E90K polymorphic status. The percent reduction in PG after sildenafil treatment was compared in (**c**) three (wildtype, heterozygous, and homozygous) groups and (**d**) two (wildtype and variant) groups. The bars and boxes show the median and interquartile ranges. The whiskers represent 1.5 times the interquartile range, and the black squares indicate outliers. *PDE5A* polymorphic status did not affect the PG before and after sildenafil treatment, and the percent reduction in PG among different genotype groups.

On echocardiographic examination prior to starting sildenafil treatment, septal flattening was noted in 7/9 (77.8%) wildtype, 6/13 (46.2%) heterozygous, and 15/19 (78.9%) homozygous dogs with no significant difference in frequency among genotype groups (*P* = 0.12). One dog in the homozygous group had signs of right-sided heart failure, and one dog in the heterozygous group had signs of both left and right-sided heart failure. There were no significant differences in the estimated PG values among different genotype groups before and after treatment. Percent reductions of PG after treatment with sildenafil were compared and there were no statistically significant differences among genotype groups (Fig. 5 and Table 2). PVAT, PVET, and PVAT:ET before and after the sildenafil treatment were compared based on the *PDE5A* polymorphic status, and there were no statistically significant differences pre- and post-treatment among genotype groups (Table 3). Percent changes in these PA flow parameters were also not significantly different among genotype groups (Table 3). When variant (heterozygous and homozygous) dogs were pooled and compared to the wildtype animals, no significant differences in pre-treatment, post-treatment, and percent changes of PG, PVAT, PVET, or PVAT:ET were noted (Tables 2 and 3).

**Table 2:** The means (±SD) or medians (IQR) of the pressure gradient before and after sildenafil treatment, and percent reduction with the treatment are shown in the different genotype groups.

		Variants			
		Wildtype	Heterozygous	Homozygous	*P*-value
PG	Pre-treatment	87.75 (±13.56)	84.35 (±23.27)		0.68
			74.67 (±20.97)	90.97 (±22.93)	0.061
	Post-treatment	55.65 (52.42–81.72)	56.72 (46.1–71.68)		0.49
			56.72 (47.63–71.6)	55.91 (39.34–86.42)	0.75
	Percent reduction	0.28 (−0.13–0.35)	0.23 (0.089–0.41)		0.98
			0.23 (0.15–0.32)	0.24 (0.064–0.72)	0.88

PG: pressure gradient.

**Table 3:** The means (±SD) or medians (IQR) of the pulmonary flow profile obtained during echocardiographic examination, PVAT, PVET, and PV AT:ET before and after sildenafil treatment, and percent reduction with treatment are shown in the different genotype groups.

		Wildtype	Heterozygous	Homozygous	P-value
PVAT	Pre-treatment	40 (35–47.5)	39 (32.5–57)	42 (34–55)	0.98
	Post-treatment	48 (29–62)	53 (48–68)	42 (37.75–57.75)	0.88
	Percent reduction	−0.15 (−0.28–0.38)	0.2 (−0.061–0.66)	0.032 (−0.17–0.28)	0.41
PVET	Pre-treatment	152.8(±14.35)	174.2 (±37.91)	156.4 (±27.8)	0.16
	Post-treatment	158 (145–197)	161 (134–193)	177.5 (150.5–186.3)	0.82
	Percent reduction	0.068 (−0.012–0.31)	−0.014 (−0.15–0.088)	−0.021 (−0.090–0.32)	0.42
PVAT:ET	Pre-treatment	0.28 (0.24–0.31)	0.24 (0.18–0.31)	0.27 (0.22–0.38)	0.7
	Post-treatment	0.24 (0.19–0.39)	0.33 (0.27–0.37)	0.23 (0.22–0.35)	0.25
	Percent reduction	−2.3 (−5.55–1.04)	−2.071 (−4.47–2.61)	0.82 (−0.51–1.032)	0.42

PVAT; pulmonary artery flow acceleration time, PVET; pulmonary artery flow ejection time, PVAT:ET; the ratio of pulmonary artery flow acceleration time to ejection time.

### QOL scores

**Figure 6:**
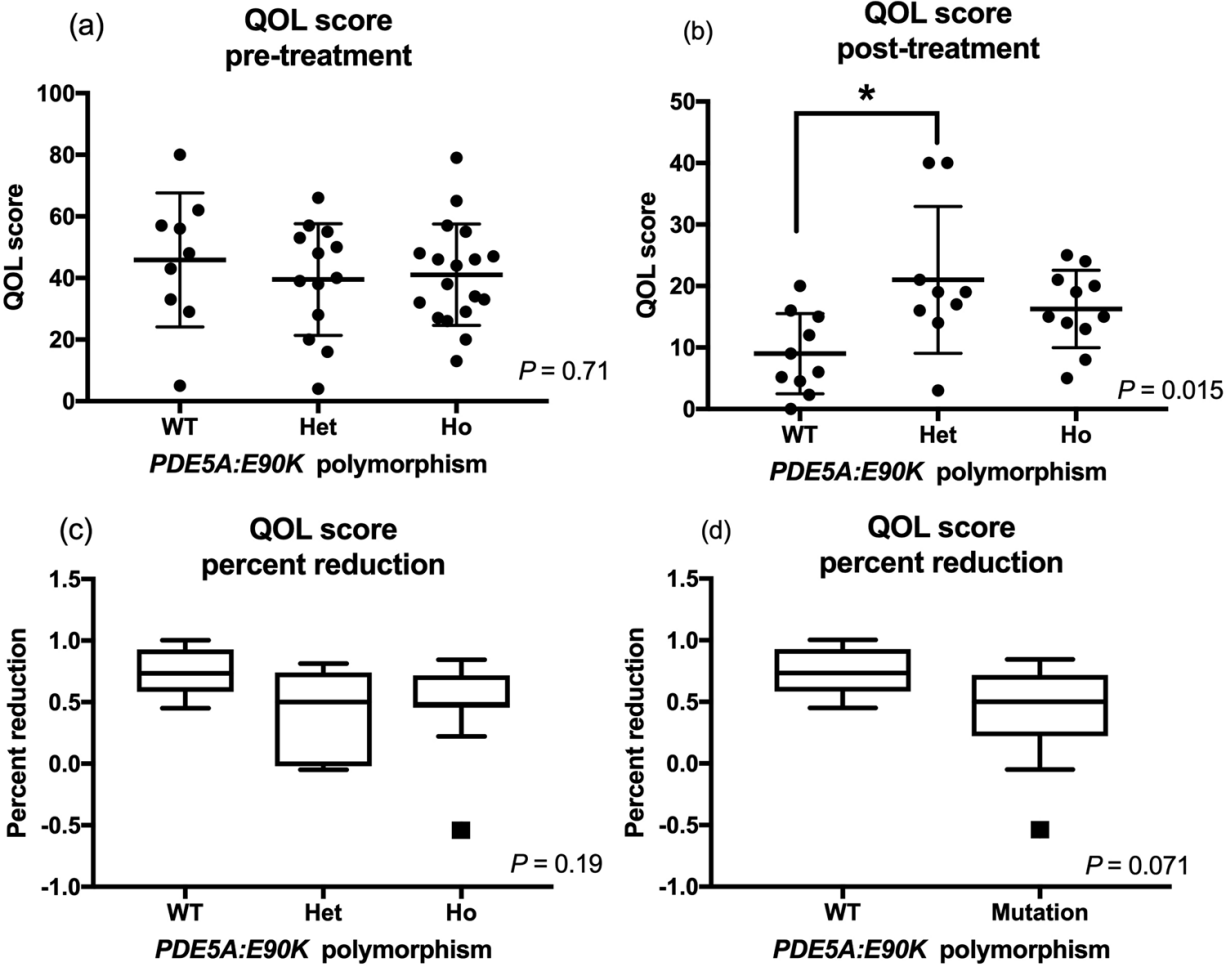
QOL scores before (**a**) and after (**b**) sildenafil treatment are shown for different *PDE5A*:E90K polymorphic status. The percent reduction in QOL scores after sildenafil treatment was compared among three (wildtype, heterozygous, and homozygous) groups (**c**) and two (wildtype and variant) groups. The bars show the mean and standard deviations with the black dots as individual values when they were normally distributed. The bars and boxes show the median and interquartile ranges when they were not normally distributed. The whiskers represent 1.5 times the interquartile range, and the black squares indicate outliers. There was a significant difference in QOL score between dogs in the wildtype and heterozygous groups after sildenafil treatment (*P* = 0.012).

Mean QOL scores prior to administering the first dose of sildenafil were not significantly different among dogs with varying genotype status, whereas there was a significant difference among the genotype groups in the 25–35 day post-treatment QOL score (P = 0.015). Post-hoc analysis revealed a significant difference in the QOL scores between dogs in the wildtype and heterozygous group (P = 0.012) after treatment (Fig. 6), although no significant difference in the percent reduction of the QOL scores was found among different genotype groups (Table 4). When variant dogs were pooled and compared to the wildtype dogs, no significant differences in pre-treatment, post-treatment, and percent changes of QOL scores were noted (Fig. 6 and Table 4).

**Table 4:** The means (±SD) or medians (IQR) of the QOL score obtained before and after sildenafil treatment, and percent reduction with treatment are shown in the different genotype groups.

		Variants			
		Wildtype	Heterozygous	Homozygous	*P*-value
QOL Score	Pre-treatment	45.89 (±21.75)	40.42 (±16.9)		0.43
			39.54 (±18.12)	41.1 (±16.5)	0.71
	Post-treatment	9 (±6.537)	18.37 (±9.57)		0.08
			21 (±11.96)	16.27 (±6.28)	**0**.**015**
	Percent reduction	0.73 (0.58–0.93)	20 (12–33)		0.071
			0.5 (−0.019–0,74)	0.48 (0.45–0.72)	0.19

QOL; quality of life.

### cGMP concentrations

**Figure 7:**
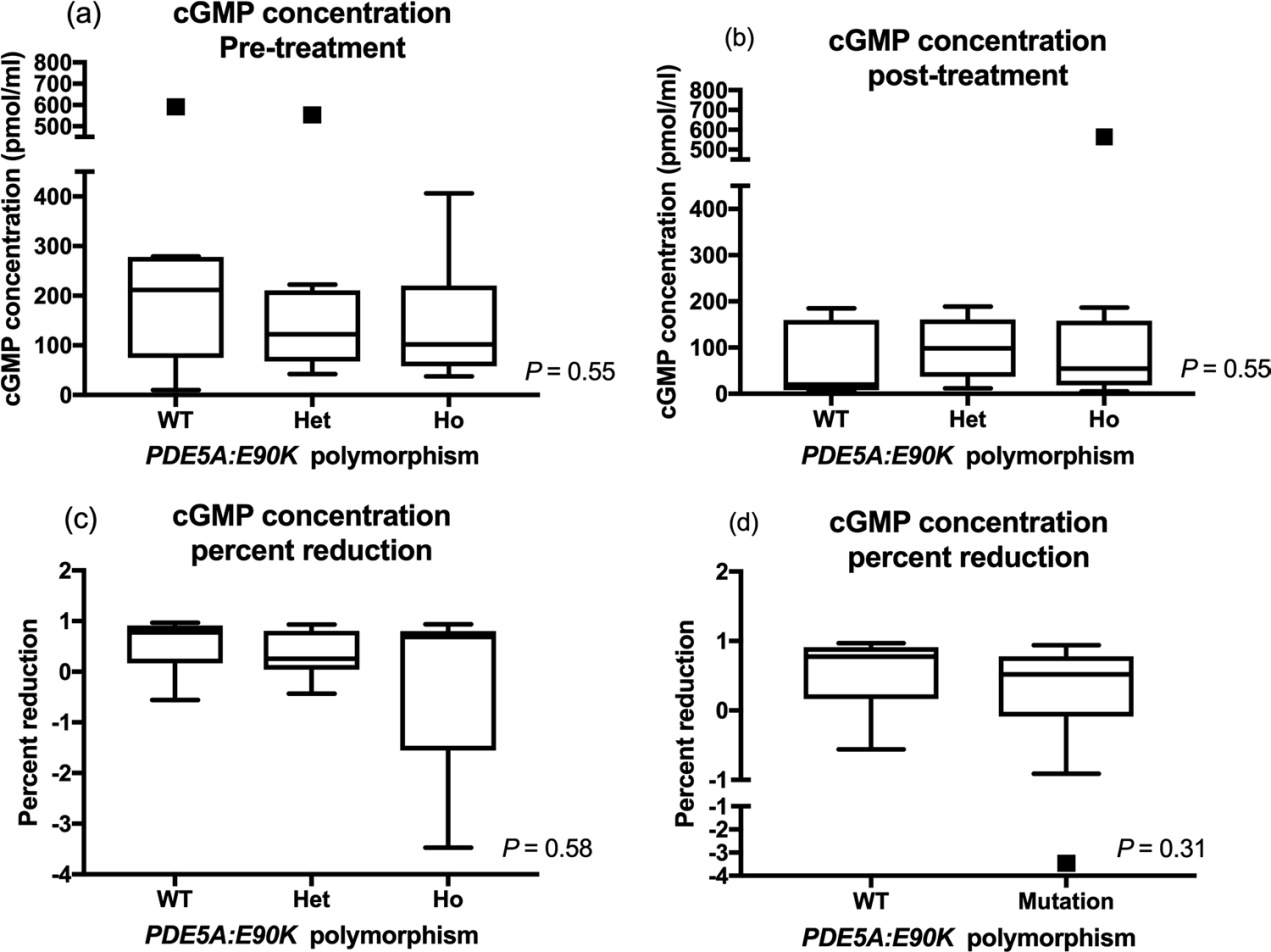
Plasma cGMP concentrations (**a**) before and (**b**) after sildenafil treatment are shown in the different *PDE5A*:E90K polymorphic status. The percent reduction in cGMP concentration after sildenafil treatment was compared in (**c**) three (wildtype, heterozygous, and homozygous) groups and (**d**) two (wildtype and variant) groups. The bars and boxes show the median and interquartile ranges. The whiskers represent 1.5 times the interquartile range and the black squares indicate outliers. There was no significant difference in the plasma cGMP concentration before and after the treatment. There was also no significant difference in percent changes of cGMP concentrations among different genotype groups.

Mean cGMP concentrations before and after sildenafil treatment were not significantly different among different genotype groups (Table 5). When percent change in the plasma cGMP concentration was compared in dogs with different *PDE5A*:E90K polymorphisms, no statistical differences among the genotype groups were noted. When variant dogs (heterozygous and homozygous) were pooled and compared to the wildtype dogs, no significant differences in the pre-treatment, post-treatment, and percent changes of cGMP concentrations were noted (Fig. 7 and Table 5).

**Table 5:** The means (±SD) or medians (IQR) of the plasma cGMP concentrations obtained before and after sildenafil treatment, and percent reduction with treatment are shown in the different genotype groups.

		Variants			
		Wildtype	Heterozygous	Homozygous	*P*-value
cGMP	Pre-treatment	211.8 (74.88–278.3)	109.3 (61.3–203.5)		0.35
			122.4 (67.51–210.7)	102 (58.02–220.6)	0.55
	Post-treatment	19.75 (8.20–159.7)	54.53 (19.5–151.7)		0.34
			98.64 (36.76–160.9)	54.53 (18.1–158.4)	0.55
	Percent reduction	0.78 (0.17–0.91)	0.52 (−0.088–0.78)		0.31
			0.25 (0.035–0.8)	0.69(−1.55–0.81)	0.58

cGMP; cyclic guanosine monophosphate.

### Predictive modelling

The predictive value of the percent reduction in the echocardiographic parameters (PG, PVAV, PVET, PVAT:ET) and plasma cGMP concentration on the percent reduction in QOL score was assessed by performing simple and multiple regression analyses. These assessments revealed the statistically significant prediction of the percent reduction in QOL score by the percent reduction in cGMP concentrations with the simple regression analysis F (1,18) = 6.35, P = 0.021, R^2^ = 0.2607. Multiple regression analysis of echocardiographic parameters and cGMP concentrations predicted the percent reduction of QOL score, F (3,16) = 4.54, P = 0.0174, R^2^ = 0.4599. Among all these predictive variables, only the percent change of cGMP added statistically significant prediction to the model (P = 0.049).

Simple and multiple regression analyses were performed to predict the effect of the independent variables (*PDE5A* genotype, dose, sex, age, the presence of septal flattening, pre-treatment blood pressure) to the dependent variables (PG, PVAT, PVET, PVAT:ET, QOL score, cGMP concentration). No variables were significantly able to predict the dependent variables by simple regression analysis when the dogs were divided up into three groups (wildtype, heterozygous, and homozygous) or two groups (wildtype and variant groups). However, multiple regression analysis showed that these independent variables significantly predicted the percent reduction of QOL scores, *F* (2, 23) = 3.67, *P* = 0.0413, *R*^2^ = 0.2421. Among all predictor variables, only genotypic status (wildtype or variant groups) added significant prediction to the model (*P* = 0.043).

## Discussion

In this study dogs with moderate to severe PH had significant reduction of PG, improvement of QOL scores, and reduction of plasma cGMP concentrations after sildenafil therapy. When these parameters were compared in dogs with different *PDE5A*:E90K polymorphism status, the QOL score was significantly worse after treatment in dogs with the heterozygous form of *PDE5A*:E90K and trended towards being significantly worse in variant genotypes when compared to wildtype dogs. The simple and multiple regression analysis revealed a significant but weak prediction for the percent reduction in QOL score by plasma cGMP and *PDE5A*:E90K genotype status after sildenafil treatment.

In the pulmonary vascular endothelium, nitric oxide (NO) is synthesized from oxygen and L-arginine catalyzed by nitric oxide synthase isoenzymes (arginine-NO pathway). NO is a potent vasodilator and an inhibitor of vascular smooth muscle cell proliferation by activating guanylyl cyclase which increases cGMP production^49,50^. Apart from the arginine-NO pathway, nitrite (NO_2_^−^) and nitrate (NO_3_^−^) also increases cGMP concentration by bio-transformation to NO via both non-enzymatic and enzymatic mechanisms (nitrate-nitrite-NO pathway)^51–53^. Circulating nitrite (NO_3_^−^) is endogenously produced from the arginine-NO pathway, and also determined by the amount of dietary intake of nitrates (NO_3_^−^). In the present study, plasma or tissue concentrations of L-arginine, nitrite, nitrate, and NO were not measured, and the dominant pathways of NO and subsequent cGMP production in these patients were not determined^54,55^. It is possible that dogs with high nitrate intake could have higher nitrite levels and subsequent cGMP production. This represents an additional possible cause of interindividual variability for sildenafil treatment response. Despite this possible confounding variable, this remains an unlikely cause of the inter-individual variability observed in this study since the baseline cGMP concentrations were not significantly different between genotype groups.

Sildenafil has been used with overall encouraging result as a treatment for canine PH^12,13,21^. One prospective and several retrospective studies demonstrated that sildenafil significantly decreased PA pressure in the majority of dogs with PH^13,21,22^. However, another study reported no significant reduction in PA pressure after sildenafil treatment despite finding a significant improvement in quality of life with sildenafil therapy^12^. In our study, PG values were significantly reduced after sildenafil treatment, but other echocardiographic parameters routinely used to assess PH severity^12^ including PVAT, PVET, and PVAT:ET were not significantly altered. In addition, the QOL score was significantly improved after sildenafil treatment in dogs with moderate to severe PH. Although no significant correlation was identified in the present study between percent changes of PG and QOL scores, it is reasonable to speculate that the observed reduction in PG is due to sildenafil treatment and is associated with improved QOL score. This is consistent with some of the previous studies of PH in humans. Interestingly, the only prospective randomized placebo controlled study in dogs with PH failed to identify any significant reduction of PA pressure between the sildenafil or placebo groups^21^. The authors of this prospective study, however, stated that this was possibility due to type II error because of withdrawal of dogs during the study period. The present study did not include a placebo control group, and thus we are not able to confirm the improvement in PG, QOL scores and cGMP concentration are entirely a result of sildenafil therapy. Although unlikely given the typical clinical progression of canine PH, the authors cannot rule out that the improvement of these parameters was partially due to improvement of the underlying disease condition during the study period. Despite these confounding variables of clinical research, in the present study, a significantly lower cGMP concentration was measured after sildenafil treatment. There was no significant correlation between the percent reductions in PG and cGMP concentration, yet there was a weak but significant correlation between the percent reductions of plasma cGMP concentration and the QOL score. These results might also suggest that the lower circulating cGMP concentration could result from improvement in PH secondary to sildenafil therapy and/or improvement of the underlying disease condition with subsequent reductions in endogenous cGMP production. While not specifically evaluated in this study, it is possible that the change in circulating cGMP might be considered as a potential biomarker for studying the response to sildenafil therapy.

It is important to consider that cGMP concentration in the pulmonary vasculature could be elevated in dogs after sildenafil treatment even with lower circulating plasma cGMP concentration, perhaps reflecting sequestration of cGMP at the site of action in vascular smooth muscle and sustained activity locally. However, previous studies showed a high degree of correlation between plasma cGMP concentration and tissue cGMP concentration in the aorta, which was maintained after treatment with a PDE inhibitor^56^. Regardless of the explanation for the observed cGMP concentrations, a significant reduction of PG and improvement in QOL score was achieved with sildenafil treatment.

When dogs were categorized into two or three groups based on their *PDE5A* genotypes, no statistically significant differences in any echocardiographic parameters (PG, PVAT PVET, PVAT:ET) before or after sildenafil treatment were noted. Although the previous study reported the basal plasma cGMP concentrations were significantly lower in healthy dogs with *PDE5A* polymorphisms, the plasma cGMP concentrations were not significantly different before sildenafil treatment among different genotype groups in this study^48^. This could be explained by overall activation of arginine-NO pathway by PH in dogs with any polymorphic status or type II error due to small sample size. More importantly, post-treatment QOL scores were significantly different among groups due to significantly worse QOL score in the heterozygous group than the wildtype group. The absolute and percent reduction in QOL scores were not significantly different among groups but there was a trend toward reduced improvement in the QOL score in variant dogs compared to the wildtype dogs. Our findings suggest that the *PDE5A* gene mutation does not significantly alter basal echocardiographic and biochemical parameters, but it alters observable QOL effects in sildenafil treated dogs with moderate to severe PH. This discrepancy among the *PDE5A* genotypes however cannot be fully explained by the effect of sildenafil on the plasma cGMP concentrations alone. The failure to observe a significant change in the homozygous genotype group may simply be due to type II error as there is a comparably small number of dogs in this group and other possible mechanisms impacting QOL score by genotype should be investigated in the future.

Sildenafil is also known to act as an anti-oxidant and could improve the clinical condition of dogs with PH by reducing the oxidative stress from PH or the underlying disease condition^26,27,57–60^. This could be a potential reason that the QOL score in dogs with PH was improved without significant improvement of PA pressure in previous studies^12^. The level of oxidative stress could be evaluated by measuring various biomarkers^61–65^, however assessments of oxidative stress were not measured as part of this clinical trial. In future studies, these oxidative stress markers could be assessed concurrently to determine the efficacy of sildenafil for PH, and to determine the impact of the *PDE5A* mutation on the efficacy of sildenafil in dogs with PH.

The findings of this study pointed out several findings of translational importance. Although there are multiple canine models of PH reported in previous studies, these only represent experimentally-induced acute PH models. Canine naturally-occurring PH has been previously reported but these studies are almost exclusively retrospective uncontrolled studies with contradictory results. Our study was a prospective controlled clinical trial with long-term sildenafil therapy for dogs with naturally-occurring moderate to severe PH, and we establish that this canine model is a reasonable choice for further experimental studies to determine long-term efficacy of medical and surgical interventions for patients with PH of various etiologies in various species. In addition, it is the first study to report associations between a *PDE5A* gene mutation and efficacy of sildenafil in dogs with PH. This finding signifies the importance of investigating the similar genetic mutations in *PDE5A* genes in human patients with PH to better understand the inter-individual variation of the efficacy of sildenafil. It further represents an opportunity for personalized medicine that could enable clinicians to make personalized clinical recommendations guided by genotype.

In the present study, there are several limitations. Based on the previous study conducted by one of the authors using apparently healthy dogs, the polymorphic genotype was expected to be common with more than 75% of the canine population possessing the polymorphism^48^. It was thus difficult to enroll the *PDE5A* wildtype dogs to fully satisfy the statistical power. Indeed, some of the values assessed, including the absolute and percent changes of cGMP concentrations before and after the treatment, were trending toward a significant difference, which may be indicative of a type II error due to small sample size. The small sample size of dogs with PH was therefore a possible cause of a failure of rejecting the null hypothesis in the present study. Future studies aiming to address these questions should thus include larger numbers of dogs with PH, especially targeting *PDE5A* wildtype and homozygous dogs to elucidate the impact of *PDE5A* gene mutation. Secondly, the present study enrolled dogs with PH caused by any underlying diseases and with concurrent diseases, which could alter plasma cGMP concentrations as well as impact response to sildenafil therapy. Although this is less likely to be a significant problem since none of the enrolled dogs had significant systemic illness other than PH and the underlying cardiopulmonary diseases, future studies may aim to enroll dogs with a more homogenous PH etiology, although this will limit the direct applicability to the clinical experience, which our study mimicked through its open enrollment. Potentially significant confounding variables include nitrite and nitrate concentrations as well as oxidative stress level due to cardiopulmonary and concurrent disease conditions, and future studies should consider measuring the concentrations of nitrate and nitrite as well as oxidative stress markers. Third, PA pressure was estimated by echocardiographic examination in this study. The gold standard for diagnosing PH is a direct measurement of PA pressure by right heart catheterization. In people, there are conflicting findings as to the correlation of PA pressure measured by echocardiography and by right heart catheterization. In clinical veterinary medicine, the right heart catheterization is not routinely used to measure the PA pressure due to its high degree of invasiveness and the requirement for anesthesia which falsely reduces measured pressure gradients. As a future direction, the long-term effect of sildenafil in dogs and the pharmacogenomic effect of the *PDE5A* mutation on interindividual-variability of sildenafil therapy could be assessed in the experimental setting through right heart catheterization and echocardiographic evaluations. Finally, in this study, cGMP concentration was measured in the plasma obtained from the dogs. Although cGMP in plasma is strongly correlated with that in the endothelium of other species, it is unknown if this strong correlation could be maintained in dogs with sildenafil treatment.

This study demonstrated that sildenafil treatment significantly reduces PG, reduces plasma cGMP concentration, and improves QOL scores in dogs with moderate to severe PH. The percent reduction in QOL score is correlated with the percent change in cGMP but not percent change in PG. The changes in echocardiographic and biochemical parameters observed with sildenafil treatment were not affected by the genotype of *PDE5A* gene, but the improvement in QOL score appears blunted in dogs with the heterozygous allele state of this mutation. Therefore, inter-individual variability in the efficacy of sildenafil as a treatment of PH could be partially explained this *PDE5A* mutation. Further studies are warranted to fully understand the association of this *PDE5A* mutation and the efficacy of sildenafil in treating moderate to severe canine PH and to further document the role of genetic testing and individualized medicine in this disease.

## Methods

### Animals and study settings

This study was approved by the Animal Care and Use Committee of the University of California-Davis and performed in accordance with relevant guidelines and regulations. Informed owner consent was obtained for all dogs prior to enrollment. All dogs were enrolled between January 2014 and August 2016. Dogs were evaluated prior to enrollment by performing a general physical examination, cardiovascular examination, complete echocardiogram, and systolic blood pressure measurement using sphygmomanometer and Doppler device at the William R. Pritchard Veterinary Medical Teaching Hospital of the University of California-Davis. Dogs were enrolled if the tricuspid regurgitation estimated systolic pulmonary pressure gradient (PG) was ≥50 mmHg in the absence of outflow tract obstruction, pulmonic stenosis, systemic hypotension (SBP < 90 mmHg) or systemic hypertension (SBP > 180 mmHg). Any medications except those that directly alter PA pressure were allowed but these medications and their doses could not be modified. Uncontrolled left-sided congestive heart failure was considered as an exclusion criterion as institution of new cardiac medications was not permitted.

The etiology of PH was categorized broadly into two basic groups: post-capillary and pre-capillary etiologies. The post-capillary group included dogs with left heart disease with and without other concurrent causes of PH. The pre-capillary group included dogs with an absence of left heart diseases (i.e., normal left atrial size) based on the echocardiographic examination^5^. After enrollment, the dog owners completed the previously validated 17 point QOL questionnaire, which assessed the presence and severity of clinical signs impacting breathing and functional status on a scale of 0 (not at all) to 5 (very much)^66^. A 4 mL whole blood sample was then obtained from dogs and aliquoted to EDTA and sodium-citrated tubes for extracting genomic DNA and for measuring cGMP concentrations, respectively. Sildenafil was prescribed to enrolled dogs at a dose range of 1.0–2.0 mg/kg orally every 8–12 hours for a period of 25–35 days. A reevaluation was performed in 25–35 days that included general physical examination, cardiovascular examination, and echocardiographic examination. A 2 mL of whole blood was obtained in a sodium-citrated tube for repeated cGMP measurement, and the dog owners completed a second and final QOL questionnaire during the recheck examination.

### Echocardiography

Complete echocardiographic examination including 2-dimensional, M-mode, and Doppler evaluations was performed on all dogs by a board-certified veterinary cardiologist (JS) or by a cardiology resident with the direct supervision of a board-certified veterinary cardiologist (JS, LV, CGH) using a 4- to 12 MHz sector-array transducer (S12–4) with color and spectral Doppler capability (Philips IE33, Philips Healthcare, Andover, MA). Dogs were placed in right and left lateral recumbency for echocardiographic examination. In accordance with the guidelines of the American Society of Echocardiography, all measurements were performed using the standard offline analysis software (Syngo Dynamics Workplace, Siemens Medical Solutions, USA, Inc., Malvern, PA) by using a leading-edge to leading-edge technique of measurement on at least three cardiac cycles^67^. The following echocardiographic parameters were recorded at baseline and 25–35 day after sildenafil therapy: PA flow velocity (PV), PA flow acceleration time (PVAT), PA flow ejection time (PVET), the ratio of PVAT to PVET (PVAT:ET), presence or absence of septal flattening during systole or diastole, presence or absence of right-sided congestive heart failure evidenced by cavitary effusion. PA flow was measured from the right parasternal short axis view using pulsed-wave Doppler imaging. Tricuspid regurgitation jet identified by color Doppler images was aligned parallel to the plane of the ultrasound interrogation cursor (Fig. 1). Peak tricuspid jet velocity was measured only when the patient was in sinus rhythm. PG was calculated using the modified Bernoulli equation (PG = 4 × velocity^2^) without adding estimated right atrial pressure. Moderate pulmonary hypertension was categorized as a PG of 50–75 mmHg while severe pulmonary hypertension was set at a PG > 75 mmHg.

### DNA sequencing

Four milliliters of whole blood were obtained from cephalic or saphenous veins. Two milliliters of whole blood were collected in an EDTA tube for isolation of genomic DNA. Genomic DNA was extracted using a commercially available kit (Puregene, Gentra Systems, Minneapolis, MN), in accordance with the manufacturer’s protocol. Briefly, red blood cells in EDTA whole blood samples were lysed with red blood cell lysis solution, followed by ten-minute incubation at room temperature and centrifugation. The white blood cell pellet was then resuspended in cell lysis solution overnight at room temperature. Samples were then placed on ice for ten minutes after the addition of one milliliter of protein precipitation solution. DNA was precipitated using 100% isopropanol, cleaned up with 70% ethanol, and resuspended in 100–200 ul of TE buffer. Genomic DNA was quantified and evaluated for quality and purity using NanoDrop One Spectrophotometer (Thermo Fisher).

Previously published primers for the *PDE5A:E90K* polymorphism in dogs were utilized for polymerase chain reaction (PCR)^48^. PCR reaction was performed with LA Taq with GC Buffer (Takara Bio USA) following the manufacturer protocol. Briefly, the PCR reaction contained 0.25 ul of LA Taq, 12.5 ul of 2x GC Buffer 1, 4 ul of 2.5 mM dNTP, 1 ul of 20 ng/ul forward primer, 1 ul of 20 ng/ul reverse primer, 1 ul of 20 ng/ul sample DNA, and 4.25 ul of ddH2O. The amplification protocol included an initial one-minute denaturing step at 94 °C, 30 cycles at 94 °C for 30 seconds, 55 °C for 30 seconds, 72 °C for two minutes, and a final elongation step at 72 °C for five minutes. Successful PCR amplification was verified by electrophoresis on a 1% agarose gel with 5 µL of each PCR product and 2 µL of 6x loading dye. Residual amplification primers and dNTPs were removed from the PCR product using ExoSAP-IT (Affymetrix, Santa Clara, CA). Amplicons for both forward and reverse primers were sequenced at the UC Davis Sequencing Facility (Davis, CA) using ABI Prism 3730 Genetic Analyzer. Sequences were analyzed using Lasergene Software (DNASTAR, Inc) to determine the presence of the *PDE5A*:E90K polymorphism. Genotypes were recorded as wild type, heterozygous or homozygous.

### cGMP measurement

A commercially available cGMP EIA kit (Alfa Aesar, Catalog No. BT-740, Tweksbury, MA) was used to measure plasma cGMP concentrations in dogs with PH. This product was validated for use in dogs by testing intra-assay precision (coefficient of variation < 10%), spike recovery, and dilutional linearity. Plasma cGMP concentration was measured for each of 41 samples in triplicate by spectrophotometry. Samples with >10% coefficient of variation were repeated. Plasma cGMP concentration was measured before and after 25–35 days of sildenafil treatment.

### Statistics

The sample size was calculated as to obtain an 80% power in identifying a statistically significant difference in response to sildenafil therapy based upon *PDE5A* polymorphism. The biological variable of interest was defined as PA pressure and the mean utilized for severely affected dogs in accordance with the literature^13,21^. The goal was to detect a difference in PG of 5–10 mmHg between *PDE5A*:E90K polymorphic status. Based on the frequencies of homozygous and heterozygous mutations in the preliminary study and an assumption of 10%, 15%, and 20% reduction of PG in homozygous, heterozygous and wildtype groups, respectively, a minimum of 13 dogs of each genotype category was needed to achieve 80% power with alpha of 0.05 and two-sided test^48^.

Normality testing was performed using D’Agostino – Pearson Omnibus testing. The echocardiographic parameters (PG, PVAT, PVET, PVAT:ET), QOL score, and cGMP concentrations were compared before and after sildenafil treatment using paired student’s t-test (parametric) or Wilcoxon matched-pairs signed rank test (non-parametric). Age, body weight, sildenafil dose, pre-treatment systolic blood pressure, mortality rate, echocardiographic parameters, QOL score, and cGMP concentrations for each different *PDE5A*:E90K polymorphism status were compared using either an ANOVA (parametric) with subsequent Tukey’s multiple comparisons test or Kruskal Wallis test (non-parametric) with subsequent Dunn multiple comparisons test for continuous variables. The same variables were also compared between the two groups, wildtype and variant (heterozygous and homozygous) groups, using either a student’s t-test (parametric) or Mann – Whitney U-test (nonparametric). Categorical variables including sex distribution, outcome (death/euthanized or alive) during the study period, and etiology of PH (pre- or post-capillary) were compared using a Chi-square or Fisher’s exact test. Percent change was determined by dividing the absolute reduction value by pre-treatment value.

Correlations and predictive values of the independent variables (age, sex, weight, sildenafil dose, the presence of septal flattening before treatment, etiology, *PDE5A* genotype and pre-treatment blood pressure) to various dependent variables (percent changes of PG, PVAT, PVET, PVAT:ET, QOL score, and cGMP concentration) were assessed by performing simple and multiple regression analyses, after standard assumptions for linear regression were performed. Backward and forward selection techniques were performed by sequentially deleting or adding the variables with P < 0.2, respectively. The predictive values of the percent changes of echocardiographic parameters and cGMP concentrations on the percent changes of QOL scores and cGMP concentrations were also assessed by performing simple and multiple regression analysis after standard assumptions of linear regression. Statistical significance was set at *P* = 0.05. All statistical analyses were performed on commercially available STATA software (Stata Corporation v14.2, College Station, TX) and GraphPad Prism software (GraphPad Prism v7.0c, La Jolla, CA).

## Data Availability

The authors declare that all data supporting the findings of this study are available within the article or from the corresponding author upon request.
